# Arrhythmogenic Cardiomyopathy: Current Updates and Future Challenges

**DOI:** 10.31083/j.rcm2506208

**Published:** 2024-06-04

**Authors:** Zafraan Zathar, Nihit Shah, Nimai Desai, Peysh A Patel

**Affiliations:** ^1^Department of Cardiology, Worcestershire Acute Hospitals NHS Trust, WR5 1DD Worcester, UK; ^2^Department of Cardiology, Royal Wolverhampton NHS Trust, WV10 0QP Wolverhampton, UK; ^3^Department of Cardiology, University Hospital Birmingham NHS Trust, B15 2GW Birmingham, UK

**Keywords:** arrhythmogenic cardiomyopathy, desmosomal genes, cardiac magnetic resonance imaging, electrophysiology

## Abstract

Arrhythmogenic cardiomyopathy (ACM) epitomises a genetic anomaly hallmarked by a relentless fibro-fatty transmogrification of cardiac myocytes. Initially typified as a right ventricular-centric disease, contemporary observations elucidate a frequent occurrence of biventricular and left-dominant presentations. The diagnostic labyrinth of ACM emerges from its clinical and imaging properties, often indistinguishable from other cardiomyopathies. Precision in diagnosis, however, is paramount and unlocks the potential for early therapeutic interventions and vital cascade screening for at-risk individuals. Adherence to the criteria established by the 2010 task force remains the cornerstone of ACM diagnosis, demanding a multifaceted assessment incorporating electrophysiological, imaging, genetic, and histological data. Reflecting the evolution of our understanding, these criteria have undergone several revisions to encapsulate the expanding spectrum of ACM phenotypes. This review seeks to crystallise the genetic foundation of ACM, delineate its clinical and radiographic manifestations, and offer an analytical perspective on the current diagnostic criteria. By synthesising these elements, we aim to furnish practitioners with a strategic, evidence-based algorithm to accurately diagnose ACM, thereby optimising patient management and mitigating the intricate challenges of this multifaceted disorder.

## 1. Introduction

Arrhythmogenic cardiomyopathy (ACM) is a genetic disorder affecting the myocardium. Initially, it was termed arrhythmogenic right ventricular cardiomyopathy (ARVC) as it was thought to predominantly affect the right ventricle (RV) with predisposition to fatal arrythmias. This is characterised histologically by progressive replacement of myocytes by fibrous tissue, with preponderance in the RV free wall in a region known as the triangle of dysplasia (between anterior part of pulmonary infundibulum, the infero-posterior wall and RV apex) [1]. These lesions typically extend from epicardium to endocardium. In light of developments in imaging, genotyping and our overall understanding of the disease, there is emerging recognition that this disease process is not exclusive to RV with left ventricular (LV) and biventricular ACM being recognised as phenotypic variants. The change in nomenclature from ARVC to ACM is a reflection of our improved understanding of this disease.

Estimated prevalence of ACM is between 1:2500 to 1:5000 but this is likely to be an underestimate as it does not include biventricular or LV dominant variants [2]. As sudden cardiac death (SCD) can be an initial manifestation of ACM, the true prevalence is likely to be higher than reported. The variation in the presentation frequently means that alternate differentials of myocarditis or dilated cardiomyopathy are initially considered before ACM is identified, often at the point of detailed imaging acquired through the use of cardiac magnetic resonance imaging (CMR).

In this review, we aim to provide a succinct overview of genetics and presentation with respect to electrophysiological characteristics. In recent years, there have been several changes and updates to the diagnostic criteria for ACM which we will summarise along with pertinent updates in imaging and management.

## 2. Genetics

ACM is a hereditary cardiovascular disorder with complex genetic underpinning. Understanding the genetic basis of ACM is crucial for accurate diagnosis, risk stratification and management of these patients.

In 2000, a study by McKoy *et al*. [3] in patients with Naxos disease revealed genetic mutations in a gene (Junctional Plakoglobin (*JUP*)) that encodes for plakoglobin (a protein that is a component of desmosomes). Desmosomes are specialised structures that facilitate cell-to-cell adhesion and are crucial for maintaining the structural integrity of cardiac tissue. Mutations in desmosomal genes disrupt these adhesion complexes, compromising the mechanical strength of myocardial cells which eventually leads to cell death and fibrofatty replacement [3]. This discovery led to focused efforts to identify other genes that code for desmosomal proteins and this identified mutations in the desmoplakin (*DSP*) gene, plakophilin-2 (*PKP2*), desmoglein-2 (*DSG2*) and desmocollin (*DSC2*) genes [4, 5, 6]. Non-desmosomal proteins have also been implicated in ACM, including those encoding for adherens junction proteins, ion channels and their modulators and cytoskeleton structures [7].

To date, there are 15 genes that have been implicated in pathogenesis and include both desmosomal genes (*PKP2*, *DSP*, *DSC2*, *DSG2* and *JUP*) and nondesmosomal genes (transmembrane protein 43 (*TMEM43*), desmin (*DES*), titin (*TTN*), phospholamban (*PLN*), and ryanodine receptor-2 (*RYR2*)) [7, 8]. Of these 15 genes, 8 genes (*PKP2*, *DSP*, *DSG2*, *DSC2*, *JUP*,* TMEM43*, *PLN*, and *DES*) have at least moderate evidence to be considered ACM causative [9]. It is important to note that many have no detectable pathological variants; these patients may reflect unknown pathological variants, or may harbour mutations in genes not currently associated with ACM [10].

Up to 50% of ACM cases are thought to be caused by mutations affecting desmosomal proteins [8], and of these mutations, the *PKP2*, encoding Plakophilin-2, is implicated in up to 20–46% of cases [7]. Plakophilin-2 plays a critical role in stabilizing desmosomes, and its mutations contribute to the breakdown of these structures initiating the pathological changes observed in ACM. Mutations in *DSP* and *DSG2* genes are thought to account for 10% of cases of ACM whilst mutations in *DSC2* account for approximately 5% of cases. Mutations in *JUP* gene are thought to account for less than 1% of cases [7]. Desmosomal mutations tend to have an autosomal dominant (AD) inheritance pattern with incomplete penetrance leading to an isolated cardiac phenotype with *PKP2* mutations being mostly AD in inheritance and most likely to lead to conventional phenotype than other desmosomal mutations [1, 11]. In a study performed by Biernacka *et al*. [12], ACM patients with *PKP2* mutations were less likely to present with LV involvement and heart failure symptoms and overall had a favourable prognosis compared to other mutations. Patients with *DSP* mutations present with variable phenotypes such as biventricular cardiomyopathy, or isolated LV arrhythmogenic cardiomyopathy with Rigato *et al*. [13] presenting higher degree of LV involvement in patients with *DSP* mutations. *DSP* mutation phenotypes of ACM have also been associated with higher risk of ventricular arrythmias and SCD [14]. Homozygous or compound heterozygous mutations in *JUP*, *DSP* and *DSC2* can lead to cardio-cutaneous syndromes such as Naxos and Carvajal syndromes [11].

Non-desmosomal genes contribute to development of ACM in several ways; *TMEM43* gene encodes for a nuclear envelope protein and mutations in this gene have been associated with high penetrance and risk of SCD especially in young men [15]. *DES* genes encode for intermediate filament desmin and have been identified in patients with ACM (with right predominant or biventricular involvement). *DES* mutations have also been linked to dilated cardiomyopathies (DCM), suggesting an overlap syndrome [16]. *TTN* genes encode for sarcomeric protein titin and have been found in high proportion of patients with ACM (18%) [11].

Filamin C, an actin binding protein (encoded by *FLNC* gene) plays a vital role in anchoring membrane proteins to cytoskeleton, thereby contributing to sarcomere maintenance. Truncating mutations in *FLNC* have been linked with phenotypically left dominant ACM with high risk for sudden cardiac death [17]

Finally, given the hereditary component to the disease, family screening and genetic counselling forms an integral part of diagnosis. Indeed, family screening has been a component of the diagnostic criteria in all its iterations [18, 19, 20]. Current guidelines recommend that first-degree relatives undergo clinical evaluation every 1–3 years with 12-lead electrocardiogram (ECG), ambulatory ECG and imaging [21]. However, disease expression of ACM is variable even in the same family or those carrying the same pathogenic mutation, therefore, Muller *et al*. [22] recently undertook a study to determine if there are predictors of disease development among at-risk relatives. In this study, symptomatic relatives, those 20 to 30 years of age and those with one minor task force criteria had higher hazard for developing ACM. This may help clinicians risk stratify patients that may benefit from more frequent follow-up.

## 3. Electrophysiological Characterisation

**Fig. 1.**
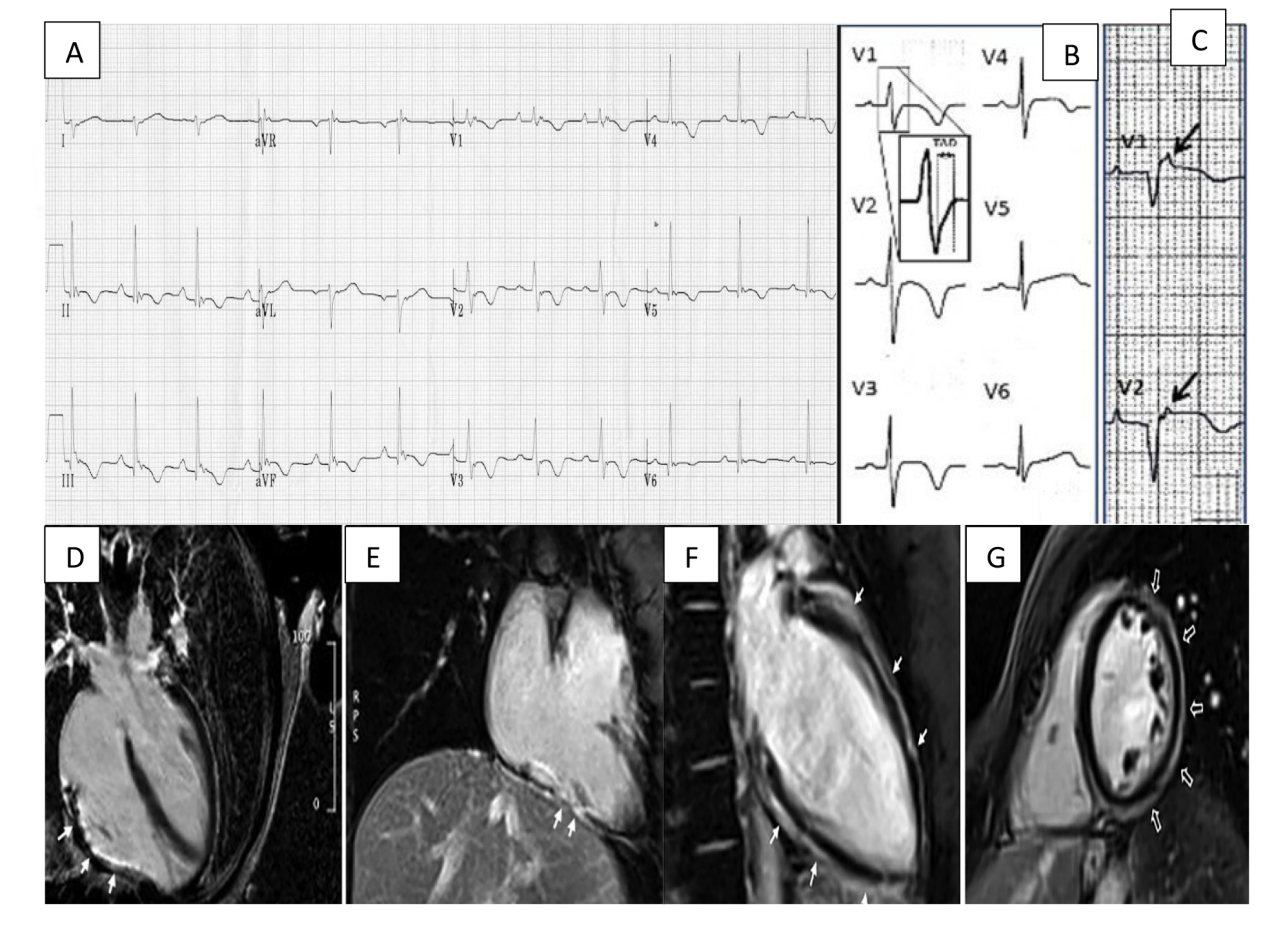
**Arrhythmogenic cardiomyopathy findings electrocardiogram (ECG) and CMR. **(A) ECG focusing on the precordial leads with characteristic T wave inversion in leads V2–V5 and an epsilon wave in V1. (B) Widening of the QRS with classical T wave inversion in leads V2–V4. A close-up view of the delayed S upstroke within the QRS is also indicated. (C) Arrows highlighting epsilon wave in V1–V2. (D) A long axis 4 chamber view CMR showing LGE/fibrous replacement of RV diaphragmatic free wall (indicated by the arrows). (E) Sagittal view CMR emphasising the fibrous replacement of the RV anterolateral wall. (F) CMR depicting subepicardial LGE of the LV extending from the base to apex segment in the sagittal view. (G) CMR image showing a post-contrast ‘ringlike’ pattern in the short axis view. CMR, cardiac magnetic resonance imaging; LGE, late gadolinium enhancement; LV, left ventricle; RV, right ventricle. Figure adapted with permission from (1) ECG Library. Life in the Fast Lane. https://litfl.com/ecg-library/. Accessed March 26, 2023. (2) Corrado *et al*. Proposed diagnostic criteria for arrhythmogenic cardiomyopathy: European Task Force consensus report. International Journal of Cardiology. 2024 Jan; 395: 131447. [20]

Understanding the electrophysiological perturbations implicated in ACM is essential for screening, diagnosis, risk stratification and guiding therapeutic interventions. The nature of structural changes within the myocardium modulates transmission of electrical signals and can result in conduction defects that may be visible on a standard 12-lead ECG (Fig. 1, Ref. [20]).

The most common ECG changes include:

∙ T-wave abnormalities: Fibrofatty replacement leads to delayed repolarisation and altered activity during the repolarisation phase (phase 3). This is represented by T wave inversion. Negative t-waves in $V_{1}$–$V_{3}$ (in the absence of right bundle branch block) is observed in RV dominant phenotypes whilst negative t-waves in $V_{4}$–$V_{6}$ (in the absence of left bundle branch block) is observed in LV dominant phenotype.

∙ Depolarisation abnormalities manifesting as a low amplitude signal which is situated between the end of the QRS and start of the T wave, most discernible in $V_{1}$–$V_{3}$. Terminal activation duration of QRS >55 ms measured from nadir of S wave to end of QRS R’ in $V_{1}$–$V_{3}$ in the absence of complete right bundle branch block.

∙ Prolonged S-wave upstroke in $V_{1}$–$V_{3}$, which reflects delayed right ventricular activation.

∙ Epsilon waves, small positive deflections at the end of QRS complex in leads $V_{1}$–$V_{3}$; indicative of delayed depolarisation in the right ventricular free wall.

∙ Low QRS voltage (<0.5 mV) in limb leads indicating LV involvement

Often, subtle ECG changes may not be visible on 12-lead surface ECG; therefore, this requires more sophisticated techniques for identification. Signal-averaged electrocardiogram (SAECG) is a non-invasive signal-processing technique to detect subtle abnormalities in the surface ECG. It can identify low-amplitude signals at the terminus of the QRS complex, referred to as ‘ventricular late potentials’. These represent delayed activation and predispose to re-entrant arrythmias. SAECG uses computerised averaging of ECG complexes during sinus rhythm to detect small amplitude signals which occur later than rapid ventricular activation. In the 2010 International Task Force (ITF) diagnostic criteria, the presence of late potentials on SAECG was considered a minor criterion for diagnosis based on studies supporting their increased sensitivity and specificity for diagnosis of ACM [18, 23]. Interestingly, in the newer 2020 ‘Padua Criteria’, SAECG are not considered part of the diagnostic work up with the authors reporting low diagnostic accuracy [19].

Additionally, invasive electrophysiological studies (EPS) can detect and provide direct visualisation and assessment of arrhythmogenic substrate. It is noted that identifying the genotype can help confer the phenotype relating to the expected location of fibrosis, e.g., variants of the lamin A/C genotype manifest substrate foci within subaortic, mid and basalseptal regions [24].

Changes that can be observed on EPS include:

∙ Delayed activation: ACM often leads to delayed electrical activation in either/both ventricles. This delayed activation can be seen in the form of prolonged endocardial activation duration.

∙ Fractionated potentials and complex electrogram (EGM) signals: These can be observed during EPS and are indicative of heterogenous conduction, which occurs when electrical impulses are slowed down or rerouted due to abnormal tissue. Fractionated EGMs are those that show multiple spikes within a single cardiac cycle, suggesting that the electrical impulse is meandering through the heart tissue rather than following the conventional path. Complex EGMs may include late potentials and local abnormal ventricular activities (LAVA), which are signals that occur after the main ventricular activation and are associated with areas of slow conduction.

∙ Low voltage areas: The presence of fibrous and fatty tissue can dampen the amplitude of electrical signals because these tissues are less electrically active than normal myocardial tissue. During an electrophysiological (EP) study, low voltage signals are used as criteria to identify areas of the myocardium which are affected [25].

∙ Ventricular tachycardia (VT): ACM is associated with ventricular arrhythmias, including VT. EPS can induce these arrhythmias to understand origin and mechanism. VT patterns in ACM can originate based on two predominant mechanisms and typically manifest at different times in the disease course. Early phase or rapid VT occurs at a younger age with the trigger being exercise. Late phase VT presents at a later point and is triggered by scar tissue. Anatomical mapping shows low voltage signals associated with fatty infiltrate and scar tissue. Early phase VT often presents with exertional exercise whilst late phase VT presents later and at rest with no neuronal triggers. Early phase VT responds to beta blockers given its neurohormonal trigger. Late phase VT may respond to catheter ablation though long-term outcome benefit is unclear.

∙ VT entrainment utilises catheters with multiple electrodes to record electrical signals across the heart, to pinpoint origin and mechanism of tachycardia. Activation mapping identifies the critical isthmus consisting of the entry, mid-isthmus and exit pathways. It is important to acknowledge the role of the epicardial approach in these studies, given the fact that arrythmogenic material is often situated in the subepicardial or middle layers of the heart muscle. This approach is not performed in all EP studies [25].

Mapping the heart’s electrical activity in three dimensions allows identification of fixed lines of conduction block associated with reentrant circuits. This approach allows construction of a 3D map that can help plan catheter ablation approaches [26]. Further work mediated through simultaneous endocardial and epicardial delineation allowed for construction of 3D structures derived from both small and large substrate areas [27].

## 4. Diagnosis

Diagnosis of ACM can be challenging as it mimics other cardiomyopathies in presentation and imaging. However, accurate diagnosis is vital as it allows early treatment initiation and cascade screening. The 2010 revised International Task Force (ITF) criteria provided an update to the Original Task Force guidelines in the diagnosis of ACM [18]. It is a scoring system containing six disease characteristics: structural alterations on imaging, tissue characterisation, repolarisation abnormalities, depolarisation abnormalities, arrhythmia and family history. In each category, there is major, minor or no criteria that can be fulfilled. A diagnosis is confirmed if 2 major, or 1 major and 2 minor, or 4 minor criteria are fulfilled from different disease categories. A criticism of the 2010 ITF criteria has been that it focused on RV phenotypic manifestations without recognising cohorts with biventricular or LV dominant variants. For instance, the major criteria for structural alteration exclusively describes RV regional abnormalities with no reference to LV structural abnormalities. Although the 2010 ITF criteria increased diagnostic yield of ACM, it cannot be applied to ACM with LV involvement [28].

In 2020, the International Expert consensus document (‘the Padua Criteria’) provided an update to diagnostic criteria to address some of the limitations in the 2010 ITF criteria [19]. This newer classification categorises ACM into three phenotypic variants: the dominant-right, biventricular and dominant-left. Although differences between these criteria have been described well elsewhere in the literature, it is important to note some key differences [29]. As endomyocardial biopsy (EMB) is invasive with potentially serious sequelae, there is less emphasis on EMB for tissue characterisation. In the 2020 ITF criteria, this is reserved for probands with negative genotyping where histology can exclude phenocopies such as cardiac sarcoidosis. Recent advancements in use of electroanatomic voltage mapping (EMV) guided EMB has been proven to be a promising tool for targeted EMB, thereby improving diagnostic yield and reducing complication rates and should be considered when EMB is being explored [30]. There is also more emphasis on the use of contrast-enhanced cardiac magnetic resonance imaging (CE-CMR) for morphological assessment and tissue characterisation. With respect to LV involvement, there is addition of new repolarisation, depolarisation and ventricular arrythmia criteria for this subgroup. Family history and genetics remain unchanged between the two criteria.

The implementation of ‘Padua criteria’ improved diagnostic yield of ACM particularly in relation to LV involvement [29]. A refinement to the 2020 criteria was recently published this year (2024 European Task Force consensus report) with changes to major criteria for diagnosis of arrhythmogenic left ventricular cardiomyopathy (ALVC) based on cardiac magnetic resonance imaging (CMR), late-gadolinium enhancement (LGE) and ECG features (Table 1, Ref. [20]). A ‘ring-like’ pattern of LGE (subepicardial or midmyocardial involvement in ≥3 Bull’s Eye segments) is now a considered a major criteria as it was highly specific for ALVC. Low voltage QRS (<0.5 mV peak to peak) in the absence of other causes was upgraded from minor to major in this update as it is rarely observed in healthy individuals and is specific to myocardial scarring in ACM [31]. There has been not been any study to date comparing the 2020 ‘Padua criteria’ with the 2024 European task force update but it is anticipated that this will improve diagnostic yield of ACM even further.

**Table 1.** **Proposed diagnostic criteria for arrhythmogenic cardiomyopathy**.

Category	RV phenotype	LV phenotype
Morpho-functional ventricular abnormalities	Major	Minor
	∙ Regional RV akinesia, dyskinesia, or aneurysm	∙ Global LV systolic dysfunction, with or without LV dilatation
	*plus* one of the following:	
	∙ Global RV dilatation	
	or	
	∙ Global RV systolic dysfunction	
	Minor	
	∙ Regional RV akinesia, dyskinesia or aneurysm of RV free wall	
Structural alterations	Major	Major
	∙ Fibrous replacement of the myocardium in ≥1 sample, with or without fatty tissue, at histology	∙ “Ring-like” LV LGE (subepicardial or midmyocardial stria pattern) of ≥3 segments (confirmed in 2 orthogonal views)
	Minor	Minor
	∙ Unequivocal RV LGE (confirmed in 2 orthogonal views) in ≥1 RV region(s) (excluding tricuspid valve)	∙ LV LGE (subepicardial or midmyocardial stria pattern) of 1 or 2 Bull’s Eye segment(s) (in 2 orthogonal views) of the free wall, septum, or both
Repolarization abnormalities	Major	Minor
	∙ Negative T waves in right precordial leads ($V_{1}$–$V_{3}$) or beyond in individuals ≥14 year-old (in the absence of complete RBBB and not preceded by J-point/ST-segment elevation)	∙ Negative T waves in left precordial leads ($V_{4}$–$V_{6}$) (in the absence of complete LBBB)
	Minor	
	∙ Negative T waves in leads $V_{1}$ and $V_{2}$ in males ≥14 year-old (in the absence of RBBB and not preceded by J-point/ST-segment elevation)	
	∙ Negative T waves beyond $V_{3}$ in the presence of complete RBBB	
	∙ Negative T waves beyond $V_{3}$ in individuals <14 year-old	
Depolarization and conduction abnormalities	Minor	Major
	∙ Epsilon wave in the right precordial leads $V_{1-}$$V_{3}$	∙ Low QRS voltages (<0.5 mV peak to peak) in all limb leads in the absence of other causes (e.g., cardiac amyloidosis, obesity, emphysema, or pericardial effusion)
	∙ Terminal activation duration of QRS ≥55 ms measured from the nadir of the S wave to the end of the QRS, including R’, in $V_{1}$–$V_{3}$(in the absence of complete RBBB)	
Arrhythmias	Major	Minor
	∙ Frequent ventricular extrasystoles (>500 per 24 h), non-sustained or sustained ventricular tachycardia of LBBB morphology with non-inferior axis	∙ Frequent (>500 per 24 h) or exercise-induced ventricular extrasystoles with a RBBB morphology or multiple RBBB morphologies (excluding the “fascicular pattern”)
	Minor	∙ Non-sustained or sustained ventricular tachycardia with a RBBB morphology (excluding the “fascicular pattern”) ∙ History of cardiac arrest due to ventricular fibrillation or sustained ventricular tachycardia of unknown morphology
	∙ Frequent ventricular extrasystoles (>500 per 24 h), non-sustained or sustained ventricular tachycardia of LBBB morphology with inferior axis (“RVOT pattern”)	
	∙ History of cardiac arrest due to ventricular fibrillation or sustained ventricular tachycardia of unknown morphology	
Family history	Major	
	∙ Identification of a pathogenic ACM-gene variant in the patient under evaluation	
	∙ ACM confirmed in a first-degree relative who meets diagnostic criteria	
	∙ ACM confirmed pathologically at autopsy or surgery in a first-degree relative	
	Minor	
	∙ Identification of a likely-pathogenic ACM-gene variant in the patient under evaluation	
	∙ History of ACM in a first-degree relative in whom it is not possible or practical to determine whether the family member meets diagnostic criteria	
	∙ Premature sudden death (<35 years of age) due to suspected ACM in a first-degree relative	
	∙ ACM confirmed pathologically or by diagnostic criteria in second-degree relative	

ACM, arrhythmogenic cardiomyopathy; LBBB, left bundle-branch block; LGE, late gadolinium enhancement; LV, left ventricle; RBBB, right bundle-branch block; RV, right ventricle; RVOT, right ventricular outflow tract. Table adapted from Corrado *et al*. Proposed diagnostic criteria for arrhythmogenic cardiomyopathy: European Task Force consensus report. Int J Cardiol. 2024 Jan doi: 10.1016/j.ijcard.2023.131447. [20] Permission obtained via Creative Commons CC-BY license.

## 5. Non-Invasive Imaging

Non-invasive imaging plays a key role in diagnosis. Both transthoracic echocardiogram (TTE) and CMR feature in the 2010 ITF criteria and the 2020 ‘Padua Criteria’ for the diagnosis of ACM. Recently, computed tomography (CT) has also emerged as a robust tool in RV assessment [32].

### 5.1 Transthoracic Echocardiography 

Transthoracic echocardiography (TTE) remains the most widely available modality to evaluate patients with suspected ACM. Despite this, RV assessment can be challenging given its complex geometry and position as well as the fact that wall motion assessment can be subtle and subjective [11]. The RV has a crescentic shape and three distinct anatomical components, the inlet, body and outlet, which cannot be simultaneously imaged in a single 2D plane [33]. Furthermore, the shape of RV means it cannot be characterised using geometric assumptions unlike the LV. The RV is also preload dependent which can lead to dynamic variations in shape and size and limit reproducibility. Finally, RV is heavily trabeculated which can impede image analysis.

As per 2010 ITF criteria, the presence of wall motion abnormalities, RV dilatation and RV dysfunction is required for a diagnosis of ACM, with the degree of RVOT dilatation and RV fractional area change (FAC) determining distinction between major and minor criteria [18]. Developments in 3D echocardiography, strain imaging as well as tissue deformation imaging (TDI) can in theory improve evaluation especially in early stages. TDI is a doppler-based imaging technique that measures velocity of myocardial tissue motion thereby allowing quantitative assessment of ventricular function. TDI can be readily performed, is relatively independent of preload and has been shown to have good reproducibility in quantification of RV function [34]. Strain imaging measures deformation or strain of myocardial fibres during the cardiac cycle. Strain imaging offers insights into myocardial mechanics, allowing assessment of regional and global contractile function. In a study by Aneq *et al*. [35], the use of longitudinal strain by speckle tracking was shown to be useful in assessment of regional and global myocardial function of both RV and LV.

### 5.2 Cardiac Magnetic Resonance Imaging

CMR has become the gold standard for adjunct imaging evaluation of patients suspected with ACM and has an additional role in serial follow-up. CMR has the ability to provide detailed tissue characterisation including wall thickness, mass, volumes, as well as regional motion, myocardial adipose content and oedema with high temporal and spatial resolution [11]. As per 2010 ITF criteria, regional RV akinesia, dyskinesia or dyssynchronous RV contraction are required to fulfil either major or minor diagnostic criteria [18]. In the updated 2020 ‘Padua Criteria’, LV regional wall abnormalities and systolic dysfunction form part of the morpho-functional diagnostic assessment [19].

CMR can detect fibrofatty infiltration of the RV; however, this has also been described in obese patients without ACM (typically in a subepicardial distribution) and therefore is a non-specific finding [36]. As per 2010 ITF criteria, fat infiltration of the myocardium counts as a diagnostic criterion if found on EMB [37]. As discussed earlier, tissue characterisation via LGE in CMR can highlight areas of myocardial scarring and fibrosis and is well described in cases of ACM (in both RV and LV phenotypes) [38]. However, LGE in itself is a non-specific finding and may not differentiate ACM from other non-ischaemic cardiomyopathies (such as myocarditis, sarcoidosis and neuromuscular dystrophies) [36]. However the extent and distribution of LGE can help differentiate between LV phenotypes of ACM and DCM, with DCM patients typically showing subepicardial or mid-myocardial LGE pattern [39]. Interestingly in the original 2010 ITF criteria, LGE was not included in the diagnostic criterion, but has been included in the 2020 ‘Padua criteria’. The 2020 ‘Padua criteria’ acknowledged that at least one morpho-functional or structural RV or LV criterion (either major or minor) must be demonstrated for a diagnosis of an ACM phenotype variant. This change has augmented the importance of cardiac imaging, and specifically of CMR given its capability of detailed morpho-functional and tissue characterisation of both RV and LV [40].

While CMR is a valuable tool in diagnosis of ACM, it is important to be aware of some limitations of the modality. Inter-observer variability in the evaluation of RV regional wall motion remains a limitation. Along with this, misinterpretation of normal variants of RV wall motion remains common. Finally, the lack of standardised protocols also limits reproducibility.

### 5.3 Computed Tomography (CT)

Cardiac CT with its excellent spatial resolution (0.5–0.625 mm) allows for delineation of myocardium from fat, with a high degree of intramyocardial fat being demonstrated in patients with ACM [41]. The use of multidetector CT (MDCT) has previously been validated in measurements of ventricular volumes in patients with congenital heart disease when compared to CMR [42]. This indicates potential capability in measurement of RV volumes. A recent study by Venlet *et al*. [43] showed that tissue heterogeneity on CT enabled differentiation between ACM and control individuals (sensitivity: 100%; specificity: 82%). Interestingly, this study also showed utility in identifying pro-arrhythmic substrate. Though not part of the typical diagnostic imaging work-up for ACM, it has been suggested to be of particular use for accurate measurement of ventricular volumes and identification of myocardial fat infiltration. CT may also be relevant in diagnosis of ACM when CMR is contraindicated (such as claustrophobia, or non CMR conditional cardiac device) or when results are inconclusive [44].

## 6. Management Strategies

### 6.1 Exercise Recommendations

At present there is no cure for ACM; instead, management is focused on risk stratification and minimising disease sequelae. Both 2017 American College of Cardiology (ACC) and 2022 European Society of Cardiology (ESC) guidelines recognise the importance of avoiding high-intensity exercise to limit disease progression and risk of ventricular arrhythmias [45, 46]. This recommendation extends to genetic carriers even in the absence of overt disease phenotype [47]. However, one of the challenges has been defining exercise intensity and translating this into clinical practice. Metabolic equivalents (METs) are a standardised way to quantify exercise intensity and high-intensity exercise is considered to require ≥6 METs [48]. Lie *et al*. [49] noted in a cohort of 173 patients that high-intensity (≥6 METs) exercise was an independent predictor of ventricular arrythmias (odds ratio (OR) 3.82, 95% confidence interval (CI) 1.33–11.04, *p* = 0.01). However, prolonged duration of exercise (>2.5 hours/week) was not a marker for ventricular arrythmia after adjustment for exercise intensity and other confounders. Patients in this study were advised to abstain from competitive sports at the point of diagnosis. As expected, patients who continued competitive sports after diagnosis were reported to have higher incidence of ventricular arrythmias [50, 51]. Interestingly, exercise is not included in the risk prediction model (https://arvcrisk.com/) [52]. Bosman *et al*. [53] assessed the added prognostic value of exercise to the risk calculator and found that it did not increment risk significantly. Therefore, the current model can be considered accurate in risk stratification even in athletes with concurrent ACM.

### 6.2 Pharmacological Agents 

Medical therapy can be categorised into management of (a) ventricular dysfunction and (b) ventricular dysrhythmias. There is a lack of robust evidence to guide management of ACM related right ventricular impairment. ACE-inhibitors, beta-blockers, aldosterone antagonists and diuretic therapy have a class IIa recommendation in 2019 Heart Rhythm Society (HRS) guidelines for symptomatic RV dysfunction [21]. In animal studies, preload reducing therapy (via furosemide and nitrates) abrogated development of RV enlargement and induction of ventricular tachycardia [54]. Therefore, 2019 HRS guidelines have a class IIb recommendation to consider using isosorbide dinitrate in symptomatic individuals [21]. Beta-blockers are mainstay of treatment for arrythmia management as they can suppress pro-arrhythmic tendency and promote ventricular remodelling. As patients with ACM tend to be younger, sotalol is favoured instead of amiodarone as it has fewer extracardiac side effects. Ventricular arrythmias or recurrent implantable cardioverter-defibrillator (ICD) shocks can be challenging for both the patient and clinician. A case series of eight patients demonstrated that flecainide, in combination with a beta-blocker, could be feasible in refractory ventricular arrhythmia [55]. A more recent observational study of 100 patients confirmed that flecainide, in combination with a beta-blocker, decreased premature ventricular contraction (PVC) and VT inducibility during programmed ventricular stimulation [56]. Flecainide use can be limited by LV ejection fraction and other agents such as mexiletine, a class 1b agent, have been used, but currently, there is absence of high quality data in this area. Catheter ablation is an alternative option for recurrent ventricular arrythmia but success is limited by multifocal substrate and potential need for epicardial approach [57]. However, a recent series by Santangeli *et al*. [58] showed that catheter ablation, in the absence of ICD, may have some promise. In this study 32 patients who declined ICD underwent endocardial and/or epicardial catheter ablation. After median follow up at 46 months there were no deaths and 26/32 (81%) were free from documented or symptomatic VT. Although this does not provide sufficient evidence to change routine practice it does highlight that catheter ablation may have feasibility as stand-alone therapy in select patients.

### 6.3 Implantable Cardioverter-Defibrillator 

SCD is a fatal disease sequelae that is preventable through ICD. Although the device procedure has associated risks (such as infection, inappropriate shocks, repeat procedures and psychological trauma from shocks) the alternative is malignant arrhythmia and potential SCD. In patients who have suffered a cardiac arrest (due to malignant arrythmia) or have hemodynamic instability with VT, this is a straightforward decision as all major guidelines have a class 1 indication for ICD [21, 45, 46]. Selection of optimal candidates for primary prevention ICD, however, has proven difficult with no universally agreed risk stratification tool. Recently, Cadrin-Tourigny *et al*. [59] was able to develop a risk stratification score (https://arvcrisk.com/) using multinational registries comprising of 528 patients with confirmed ARVC. This model was able to predict the incidence of ventricular arrythmia with optimism-corrected C-index of 0.77 (95% CI 0.73–0.81). When compared to the 2015 International Task Force Consensus (ITFC) statement for treatment of ACM, this new model was able to provide the same level of protection with 20.3% fewer ICD implants [60]. It is noteworthy that in the prediction tool by Cadrin-Tourigny *et al*. [59] nearly half of the patients had *PKP2* pathogenic mutation and more than 90% of the participants were of Caucasian ethnicity, which is not entirely reflective of the prevalence of this disease. In patients suitable for ICD therapy, the efficacy and safety of transvenous versus subcutaneous ICD has not been fully explored. In a recent analysis of matched subcutaneous versus transvenous ICD recipients, the subcutaneous group had more inappropriate shocks whilst the transvenous group had higher procedural complications [61]. Another important consideration around subcutaneous ICD is their inability to perform anti-tachycardia pacing (ATP) which can often terminate arrhythmias painlessly and avoid need for subsequent shocks.

### 6.4 Left Ventricular Involvement

As LV predominant ACM constitutes a smaller cohort of patients, the management of this subtype is less well defined. It can commonly be misdiagnosed as other cardiac muscle disorders leading to delay in treatment initiation. Management of LV impairment in ALVC is with guideline-directed medical therapy based on ejection fraction [62]. Angiotensin-converting enzyme inhibitor, beta-blocker, mineralocorticoid receptor antagonist and SGLT2 inhibitors are referred to as the ‘four pillars’ of medical management for heart failure with reduced ejection fraction (ejection fraction ≤40%). This is complemented by treating symptoms of congestion with diuretics, fluid restriction, dietary modifications and device therapy (ICD or cardiac resynchronisation therapy). A key challenge going forwards in the ALVC subgroup is related to primary prevention ICD; there is no validated risk stratification tool for this subgroup. In the 2015 ITFC consensus statement, severe LV impairment with LV ejection fraction ≤35% was considered a high risk phenotype in ACM that would have a class 1 indication for ICD implantation [60]. Moderate LV dysfunction (LV ejection fraction 36%–45%) or moderate biventricular dysfunction is one of the major risk factors along with syncope and non-sustained VT that has a class 2 indication for ICD in the 2015 ITFC statement. The 5-year risk score by Cadrin-Tourigny *et al*. [59] did not include left ventricular involvement in their prognostication. A study looking at CMR phenotypes noted that patients with LV-dominant and biventricular presentation of ACM had worse prognosis than “traditional” lone RV phenotype [63]. In this study, LV involvement did not change the 5-year risk score; however, it was an independent predictor of the combined end points of SCD, aborted arrest and appropriate ICD therapy. It is plausible that ALVC is more sinister than ARVC and these patients may benefit from closer surveillance and earlier intervention.

## 7. Future Challenges 

The recent scrutiny and updates to diagnostic parameters meant that our inclusion criteria has changed redefining the nature of ACM. This improved phenotypic understanding of ACM provides several avenues that require future consideration including risk stratification (for the biventricular and LV phenotypes), earlier detection and pharmacological therapies to target underlying disease mechanism.

Earlier detection and diagnosis is a challenge as most patients are identified as part of a cascade screening process or after life threatening arrythmia. Although CMR provides excellent tissue characterisation and aids diagnosis, it is challenging to decide who should undergo this investigation as it is not universally accessible and can be expensive. One option to navigate the latter issue would be the use of artificial intelligence (AI) and machine learning into CMR analysis to enhance speed and accuracy of image interpretation and risk assessment [64]. Another benefit of CMR is the ability to detect myocardial scar. LV scar burden has been noted to be a predictor of arrhythmogenesis and ICD therapy in observational studies [65]. In a recent study by Gutman *et al*. [66], 452 patients with non-ischaemic cardiomyopathy and LV ejection fraction ≤35% who fulfilled criteria for primary prevention ICD were stratified according to LV scar burden to assess mortality benefit. In this study, the mortality benefit was higher in patients with primary prevention ICD and LV scar (hazard ratio 0.4; 95% CI 0.21–0.76). Although this has not been explored in the context of ALVC, with the increased use of CMR in the diagnostic pathway, this could be a valuable tool in patient selection for ICD therapy in ACM that should be explored.

Given the genetic basis for ACM, there is an appreciation that gene therapy may serve as a potential option for treatment. A mouse model was generated with the *PKP2* mutation to replicate the ACM features [67]. Adeno-associated viral therapy containing the corrected gene variant *AAV-PKP2* was found to prevent desmosomal pathological deficits. Other successful endpoints including resolution of CMR changes and length of survival were associated with *AAV-PKP2* gene therapy.

The role of cytokines, particularly *TGFβ3*, has been explored in the context of ACM [68]. Mutations in the *TGFβ3* gene, particularly in non-coding regions, have been associated with ACM, suggesting mechanisms involving paracrine and autocrine signalling. Likewise, molecular targets such as Glycogen synthase kinase-3 (GSK-3) have been explored given their role in cardiac electrophysiology [69]. In the context of ACM, this protein when inhibited in murine models mediated an improvement in histology, arrythmia burden at rest and on exertion [70]. This emphasises an area that has not been investigated thoroughly and may serve as a platform for future interventional therapy.

In relation to symptom management, bilateral cardiac sympathetic denervation (BCSD) has been used in the context of refractory ventricular arrythmia for its antiarrhythmic effect [71]. There is emerging evidence from small studies that this approach can be used in the context of ACM to reduce ICD shocks or sustained VT [72]. At present, this is a treatment option only for refractory cases with failed conventional therapy and is available in a few select centres with unclear long-term benefit.

## 8. Conclusions

ACM is a hereditary cardiomyopathy that has deleterious consequences as it may present with life threatening arrythmias and sudden cardiac death. Over the last few years, our improved understanding of the disease process has enabled refinement of the diagnostic process, allowing categorisation based on phenotypic manifestations (RV, LV or bi-ventricular predominance). There have been several updates to the diagnostic criteria for ACM which we have summarised in this review, including discussion on imaging techniques and risk stratification. We have explored current therapeutic options including pharmacological management and device therapy. Although gene therapy and immune modulation show early promise, it is still in its infancy and formal trial data is lacking.
